# Carbapenem-Resistant Enterobacterales in Long-Term Care Facilities: A Global and Narrative Review

**DOI:** 10.3389/fcimb.2021.601968

**Published:** 2021-04-23

**Authors:** Hsin-Yu Chen, Shio-Shin Jean, Yu-Lin Lee, Min-Chi Lu, Wen-Chien Ko, Po-Yu Liu, Po-Ren Hsueh

**Affiliations:** ^1^ Division of Infectious Disease, Department of Internal Medicine, Taichung Veterans General Hospital, Taichung, Taiwan; ^2^ Department of Emergency, School of Medicine, College of Medicine, Taipei Medical University, Taipei, Taiwan; ^3^ Department of Emergency Medicine, Department of Emergency Medicine and Critical Care Medicine, Wan Fang Hospital, Taipei Medical University, Taipei, Taiwan; ^4^ Department of Internal Medicine, Changhua Christian Hospital, Changhua, Taiwan; ^5^ Division of Infectious Diseases, Department of Internal Medicine, China Medical University Hospital, Taichung, Taiwan; ^6^ Department of Microbiology and Immunology, School of Medicine, China Medical University, Taichung, Taiwan; ^7^ Department of Internal Medicine and Center for Infection Control, College of Medicine, National Cheng Kung University Hospital, Tainan, Taiwan; ^8^ Department of Medicine, College of Medicine, National Cheng Kung University, Tainan, Taiwan; ^9^ Rong Hsing Research Center for Translational Medicine, National Chung Hsing University, Taichung, Taiwan; ^10^ Ph.D. Program in Translational Medicine, National Chung Hsing University, Taichung, Taiwan; ^11^ Division of Infectious Disease, Department of Internal Medicine, National Taiwan University Hospital, National Taiwan University College of Medicine, Taipei, Taiwan; ^12^ Department of Laboratory Medicine, National Taiwan University Hospital, National Taiwan University College of Medicine, Taipei, Taiwan

**Keywords:** Enterobacteriaceae, long-term care facilities, oxacillinase, carbapenemases, metallo-beta-lactamase

## Abstract

The emergence of carbapenem-resistant Enterobacterales (CRE) has become a major public health concern. Moreover, its colonization among residents of long-term care facilities (LTCFs) is associated with subsequent infections and mortality. To further explore the various aspects concerning CRE in LTCFs, we conducted a literature review on CRE colonization and/or infections in long-term care facilities. The prevalence and incidence of CRE acquisition among residents of LTCFs, especially in California, central Italy, Spain, Japan, and Taiwan, were determined. There was a significant predominance of CRE in LTCFs, especially in high-acuity LTCFs with mechanical ventilation, and thus may serve as outbreak centers. The prevalence rate of CRE in LTCFs was significantly higher than that in acute care settings and the community, which indicated that LTCFs are a vital reservoir for CRE. The detailed species and genomic analyses of CRE among LTCFs reported that *Klebsiella pneumoniae* is the primary species in the LTCFs in the United States, Spain, and Taiwan. KPC-2-containing *K. pneumoniae* strains with sequence type 258 is the most common sequence type of KPC-producing *K. pneumoniae* in the LTCFs in the United States. IMP-11- and IMP-6-producing CRE were commonly reported among LTCFs in Japan. OXA-48 was the predominant carbapenemase among LTCFs in Spain. Multiple risk factors associated with the increased risk for CRE acquisition in LTCFs were found, such as comorbidities, immunosuppressive status, dependent functional status, usage of gastrointestinal devices or indwelling catheters, mechanical ventilation, prior antibiotic exposures, and previous culture reports. A high CRE acquisition rate and prolonged CRE carriage duration after colonization were found among residents in LTCFs. Moreover, the patients from LTCFs who were colonized or infected with CRE had poor clinical outcomes, with a mortality rate of up to 75% in infected patients. Infection prevention and control measures to reduce CRE in LTCFs is important, and could possibly be controlled *via* active surveillance, contact precautions, cohort staffing, daily chlorhexidine bathing, healthcare-worker education, and hand-hygiene adherence.

## Introduction

The emergence of antimicrobial resistance has become a major public health concern. Since the identification of carbapenem-resistant Enterobacterales (CRE) in the 1990s, CRE has spread worldwide during the past two decades (Centers for Disease Control and Prevention, 2013). The threat is not only confined to tertiary referral hospitals or academic health science centers. In a network of community hospitals in the southeastern United States, a CRE incidence of 0.26 per 100,000 patient-days in 2008 and 1.4 per 100,000 patient-days in 2012 was reported (Thaden et al., 2014). In a population-based study in seven states in the United States, CRE incidence of up to 2.93 per 100,000 persons (95% Confident Interval 2.65–3.23) was also reported (Guh et al., 2015). In addition, previous studies had reported high mortality rates among CRE-infected patients, ranging from 32.1%–48% (Patel et al., 2008; Gasink et al., 2009), which could even increase to 71% in one year among liver transplant patients (Kalpoe et al., 2012). Regarding the median cost of CRE infections with an incidence of 2.93 per 100,000 persons in the United States, it would cost hospitals $275 million, third party payers $147 million, and the society $553 million (Bartsch et al., 2017), indicating a high economic burden caused by CRE infections. Collectively, considering the rapid worldwide spreading of CRE, the association of CRE infections with poor clinical outcomes, high economic burden, and relatively limited antimicrobial treatment for CRE in the current era, the Centers of Disease Control and Prevention (CDC) announced CRE as the most urgent public health threat in 2013.

During the past decade, the demand for chronic rehabilitation and skilled nursing care after an acute illness has increased as the population ages. At the same time, a high prevalence of colonization by multi-drug resistant organisms (MDRO) among residents in long-term care facilities (LTCFs) was reported. The SHIELD Orange County Project demonstrated an MDRO prevalence of 65% among nursing homes (NHs) residents and 80% among long-term acute care hospital (LTACs) residents (Mckinnell et al., 2019). Furthermore, Guh et al. reported that most CRE isolates came from individuals with a history of health care exposure or hospitalization within one year, and most CRE hospitalized cases resulted in a discharge directly to a long-term care facility or LTAC hospitals (Guh et al., 2015). Another case-control study using the Illinois hospital discharge database reported that the CRE carriage rate at the time of admission was highly associated with prior health-care facility exposure (particularly LTACs) (Lin et al., 2019). That is, compared with the relatively low prevalence rate in the community (Prabaker et al., 2012) and in the acute care hospitals (ACHs), the high prevalence of CRE in LTCFs is an important public health issue and a critical component of large-scale antibiotic stewardship programs.

Tracing back, a systematic review and meta-analysis was conducted to highlight the importance of CRE in hospital settings (Van Loon et al., 2018). In this article, we conducted a literature review to summarize the current understanding of CRE in LTCF settings, and to demonstrate that the CRE colonization and/or infection rates are truly higher among LTCFs in different geographic regions.

We searched the English-language medical literature using PubMed/MEDLINE from 1990 to 2020, using the following keywords: carbapenem-resistant Enterobacteriaceae, carbapenem-resistant Enterobacterales, long-term care facilities, nursing home, long-term acute care hospitals. The references of articles found using this search were also reviewed to identify other potential studies that were not located using the search terms. Studies reporting CRE carriage and/or infection among elderly residents or patients who lived in or being admitted from long term care facilities were reviewed. Studies that provided data on previous long term care facilities exposure in the setting of acute care hospital or community or outbreak were also included. Exclusion criteria included studies that only documenting CRE carriage and/or infection in acute care hospital, tertiary medical centers, ICU or community; studies that tested for multiple-drug organism carriage and/or infection but did not evaluate for CRE; or studies that evaluated CRE carriage and/or infection among pediatric patients or health care workers. In total, 33 studies were reviewed in full. Other 12 articles involved in this study were searched while reviewing the similar articles in above mentioned papers.

### Prevalence and Incidence of CRE Among Residents in LTCFs

An increasing number of studies have evaluated CRE in LTCFs between 2012 and 2020. In most studies, LTCF were facilities that provide long-term rehabilitation and skilled nursing care, such as long-term acute care hospitals or facilities (LTACs), skilled nursing facilities (SNFs) and nursing homes (NHs) in the United States, and residential care homes for the elderly (RCHE) in Hong-Kong. The study designs for CRE acquisition among LTCF residents could be broadly classified into four types: point prevalence survey (identifying the number of people with CRE at a specific point in time), incidence surveillance (determining the rate of CRE in LTCF for a specific period), outbreak investigations (reports of investigation during CRE outbreak), and network model formulation. CRE colonization and/or infection in LTCFs shared similar but not identical characteristics in different geographic regions; therefore, we separated the studies geographically, that is, the United States, Europe (including Turkey, Israel, and North Lebanon) and Asia.

The prevalence rate of CRE colonization among LTCFs in the United States varied widely, as demonstrated in **Table 1**, ranging from 1%–30.4% (Prabaker et al., 2012; Lin et al., 2013; Johnson et al., 2014; Van Duin et al., 2014; Cunha et al., 2016; Mckinnell et al., 2016; Prasad et al., 2016; Han et al., 2017; Reuben et al., 2017; Mckinnell et al., 2019). The prevalence rate of Carbapenem-resistant *K. pneumoniae* (CRKP) among residents of LTCFs in the United States demonstrated a high geographical variation, with the highest prevalence in the West (42.2%), followed by the South (12.2%), Midwest (7.3%), and Northeast (9.9%) (Han et al., 2017). The highest CRKP prevalence was found in California (45.5%, 701/1540) (Han et al., 2017). Among the CRKP endemic West region, the CRKP prevalence rate changed significantly across the study period: 48.5% in quarter 1, 2014; 40.4% in quarter 2, 2014; 49.5% in quarter 3, 2014; 32.5% in quarter 4, 2014; and 39.0% in quarter 1, 2015 (p<0.01, test for trend) (Han et al., 2017). The CRE incidence among residents of LTCFs in the United States varied from 1.07–6.83 cases per 10000 patient-days (Brennan et al., 2014; Chopra et al., 2018). The pooled mean incidence rate of CRE in the United States was 0.46 per 1000 patient-days (Marquez et al., 2013).

**Table 1:** Studies of carbapenem-resistant Enterobacterales colonization in long-term care facilities in the United States.

Reference	Study type	Sites	Study period	Study populations	Specimen sources	Sample size	Prevalence/incidence	LTCF percentage	Molecular studies	Risk factors
Lautenbach et al., 2009	Point prevalence study	New Jersey, Pennsylvania Delaware	2008/01/15 -2008/11/15	63 LTCFs across 3 US states	Urine culture	1,805 isolates	6% CR-KP	1,653 isolates from 44 SNF 152 isolates from 19 ALF	NA	NA
Lin et al., 2013	Point Prevalence study	Chicago	2010/07-2011/06	24 ACHs 7 LTACs	rectal, inguinal swab, or urine	391 patients	30.4% (119 of 391) of LTACHs with KPC-producing Enterobacterales, compared to 3.3% (30 of 910) of ACHs ICU patients [prevalence ratio 9.2, 95% CI 6.3-13.5]	All LTACHs had KPC, prevalence range, 10%–54%) 15 of 24 ACHs had KPC (prevalence range, 0%–29%).	NA	LTACH facility type, mechanical ventilation, and length of stay
Johnson et al., 2014	Point Prevalence study	Maryland	2010/07-2010/08	30 (67%) ACHs and 10 (83%) LTCFs	peri-anal and sputum	390 patients, total 358 samples	6% of patients (ACH/LTCF) with KPC-producing Enterobacterales.	55% (n=11) in LTCF 45% (n=9) in ACH	15 *K. pneumoniae* (60% ST258) 6 *E. Coli* (50% ST131) 1 *E. Cloacae*. Of all 19 KPC-2 3 KPC-3.	Mechanically ventilated
Han et al., 2017	Point Prevalence study	USA	2014/01-2015/03	3,470 patients across 64 LTACs	Blood, respiratory, urine	3,846 unique quarterly *K. pneumoniae* clinical cultures in 3,470 patients	24.6% CRKP (946/3846)	NA	NA	Geographic variation
Prasad et al., 2016	retrospective Point Prevalence study	New York	chart review	a single center–affiliated LTCF	rectal swab	301 residents (80-bed ventilator unit)	18.9% asymptomatic rectal CRE colonization (n=57 patients 61 isolates)	CR-KP n=46 CR *E. coli* n=15	CR-KP (n=46) 30 KPC-3 16 KPC-2 CR-*E coli* 11 KPC-3 4 KPC-2 10 additional CTX-M. [9 KP KPC-3 1 E.Coli KPC-2)	Recent CDI Tracheostomy collar mechanical ventilation
Mckinnell et al., 2016	Retrospective point prevalence study	Southern California	2015/06 - 2015/08	605 residents in 3 NHs	axilla/groin swabs	1,800 swabs from 605 residents	1% CRE	NA	NA	history of MDRO, care needs, incontinence, and catheters.
Reuben et al., 2017	Retrospective prevalence study	Washington, District of Columbia	2016/01/11-2016/04/14	8 ACHs, 2 LTACs 5 SNFs 1 inpatient rehabilitation facility	perianal swab	1,022 completed tests	5.2% CRE (n=53, 95% CI, 3.9%–6.8%)	ACH 5.0% LTCF 7.0% inpatient rehabilitation facility 0%.	4.3%(n=44) KPC 1 NDM 1 both KPC and OXA48.	NA
Mckinnell et al., 2019	Retrospective point prevalence study	Southern California	2016/09-2017/03	18 NHs and 3 LTACHs [SHIELD Orange County Project]	bilateral axilla/groin and peri-rectal swabs	50 adults in 18 NHs and 3 LTACHs	67% (n=701) MDROs 3% (n=31) CRE	NA	NA	Gastrointestinal device (OR 19.7, 95% CI 3.5-109.4, p<0.001) Prior MDROs
Prabaker et al., 2012	Hospital admission Case control	Chicago	NA	Hospitalized adults from 4 hospitals with an early KPC epidemic.	Rectal swab	180 patients from LTCF	8.3% (n=15) of LTCF had KPC-producing Enterobacterales colonization	0 (0%) of the community patients (P<.001).	NA	LTCF subtype
Van Duin et al., 2014	Hospital admission	Northeastern Ohio	2011/12/24 -2013/03/01	Hospitalized patients from LTCF and community (28%)	NA	251 patients admitted to 18 hospitals	CRKP infection in 45% patients	NA	88 CRKP isolates belong to ST258	NA
Cunha et al., 2016	Hospital admission Case-Control	Providence, Rhode Island	2012/07-2012/09	hospital admission from NHs	Fecal carriage (rectal swab PCR)	404 patients with 500 hospital admissions	4.6% CPE fecal carriage rate (n=23),	NA	2 KPC producing *Citrobacter freundii*	Gastrostomy (p=0.04)
Brennan et al., 2014	Incidence surveillance	Michigan	2012/09/01-2013/02/28	17 ACHs 4 LTAC	NA	102 cases over 957220 patient days	1.07 cases per 10,000 patient days	5% hospital onset, 65% community onset (75% had health care exposure within 90d)	89 cases *K. pneumoniae* (87.2%) 13 cases *E. coli* (12.7%)	surgery in 90 days, recent infection/colonization with a multidrug-resistant organism, recent exposures to antimicrobials
Chopra et al., 2018	Incidence surveillance Retrospective Cohort	Detroit	2011/01/01-2012/07/31	A 77-bed LTAC in Detroit	NA	30 patients with CRE 24(80%) infection, 6(20%) colonization	Incidence 6.83 episodes per 10,000 inpatient-days (30/351112)	23 (77%) patients had CRE following LTAC admission	8 CRE isolates had *bla*_KPC-3_, and belonged to ST258	
Bower et al., 2020	point incident survey	8-county Atlanta metropolitan area	2016	Georgia Emerging Infections Program (EIP), Facility-specific Connectivity Using Medicare Data	NA	NA	283 incident CRE cases	50% in ACH (n=141), 40% in SNFs (n=113) 10% in LTACHs (n=29)	CRE infections originate from almost all ACHs and half of SNFs.	Medicare patient transfers strongly correlated with CRE case-transfer data in ACHs (r=0.75; P<0.01) and LTACHs (r=0.77; P=0.03), but not in SNFs (r=0.02; P=0.85).
Marquez et al., 2013	Incidence and outbreak surveillance	NA	2010/06/01-2011/05/31	ACH, LTAC, NH outbreak in 1 LTAC	NA	814 reports (CRKP positive)	ACH (57%, n=387), LTAC (34%, n=231), SNH (8%, n=57)	pooled mean incidence rate in ACHs and LTACs was 0.46 per 1000 patient-days the rate in LTACs (2.54 per 1000 patient-days) was higher than that in ACHs (0.31 per 1000 patient-days, p<0.001)		
Endimiani et al., 2009	Outbreak surveillance	South Florida	2008/03/21 -2008/04/20	1 LTAC	NA	10 KPC-KP in 241 KP isolates (4.1%)	NA	7 KPC-KP from a LTAC (3 from hospitals)	3 KPC-KP belong to ST258	NA
Dubendris et al., 2020	Outbreak surveillance (Case control)	North Carolina (USA)	2016/10/22-2017/11/30	3 LTCF during outbreak	rectal swab	83 isolates from 76 patients	7 CROs (8.4%), 4 CRE (4.8%)	6 in LTCF (7.2%)	4 IMP (in same LTCF)	

ACHs, acute care hospitals; ALF, assisted living facility; CDI, Clostridium difficile Infection; CRO, carbapenem-resistant organisms; CPE, carbapenemase producing Enterobacterales; CRE, carbapenem-resistant Enterobacteriaceae; CRKP, carbapenem-resistant Klebsiella pneumoniae; ICU, intensive care units; KP, Klebsiella pneumoniae; KPC, Klebsiella pneumoniae carbapenemase; LTACs, long-term acute care hospitals; LTCFs, long-term care facilities; MDROs, Multidrug-Resistant Organisms; NA, not available, NDM, New Delhi metallo-β-lactamase; NHs, nursing homes; SNF, Skilled nursing facility.

In Europe (including Turkey, Israel, and North Lebanon), CRE in LTCFs also demonstrated a high geographic variance (**Table 2**). Recent studies about CRE in Europe were few and most of them reported low CRE prevalence rates among residents of LTCFs. There was a 0.3% CRE prevalence rate in LTCFs in Switzerland (37/12423) (Kohler et al., 2018), and only one resident with CRE carriage was found in each LTCF in Belgium and the Netherlands (Latour et al., 2019; Van Dulm et al., 2019). Even though a low prevalence rate was noted, the high association of CRE colonization with LTCF was still noted from the hospital admission data in Spain, reporting that about 37% of cases were health-care associated, of which 42% were nursing home residents (Palacios-Baena et al., 2016). Different from mainland Europe, a higher prevalence of CRE carriage was found among residents in LTCFs in Israel (12%) (Ben-David et al., 2011) and North Lebanon (1.7%) (Dandachi et al., 2016). Though a relatively low prevalence rate of CRE colonization in LTCFs was found in Belgium, the Netherlands, and Switzerland, high CRE carriage rates (28.4%) were reported among a LTAC rehabilitation facility (LTACRF) in central Italy, which is a CRE endemic region since 2010 (Ambretti et al., 2019).

**Table 2:** Studies carbapenem-resistant Enterobacterales colonization in long-term care facilities in the Europe.

Authors	Study type	Sites	Study period	Study populations	Specimen sources	Sample size	Prevalence/incidence	LTCF percentage	Molecular studies	Risk factors
Ben-David et al., 2011	Point prevalence study	Israel	NA	1,144 patients in 12 PACFs	Rectal swab	CRKP carriage in 1,044 patients	12% CRKP (1004/1144)	NA	NA	Prolonged length of stay, sharing a room with known carrier, antibiotic use in prior 3 months, prior culture grew CRKP
Kohler et al., 2018	Point prevalence study	Switzerland	2007/01-2017/10	NH	Urogenital, skin, other	16,804 samples from 9,940 residents	0.3% CRE (37/12,423)	NA	NA	Non-urogenital isolates, geographic
Latour et al., 2019	Point prevalence study	Belgium	2015/06 -2015/10	51 randomly selected residents per NH	Rectal swab	1,447 residents from 29 NHs	CRE carriage in only 1 resident	NA	NA	NA
Van Dulm et al., 2019	Point prevalence study	Netherlands	2014/11 -2015/08	12 LTCFs	Rectal swab	385 residents from 12 LTCFs	CRE carriage in only 1 resident	MDR-GNB carriage rate 18.2% (range 0-47%)	NA	NA
Dandachi et al., 2016	Point prevalence study	North Lebanon	2013/12-2014/04	2 NHs	Fecal swab	178 isolates from 68 NH residents	1.7% CRE (3/178)	NA	1.7% co-producers of OXA-48 and ESBL	Recent antibiotic use
Palacios-Baena et al., 2016	Hospital admission	Spain	2013/02/01-2013/05/01	in 34 hospitals	NA	NA	379 isolates from 245 patients had CPE 164(66.9%) infection 81 colonized 23 asymptomatic bacteremia	Healthcare associated in 91 cases (37%), of which 42% NH residents	Of 35 NH cases 32 *K. pneumoniae* (31 OXA-48, 1 VIM-1) 3 *E. coli* (OXA-48)	NA
Legeay et al., 2019	Outbreak surveillance	Western France	2014/05-2017/07	10 isolates from 3 intra-NH outbreak	Urine Rectal swab	NA	10 CRE in 3 outbreaks	NA	10/10 OXA-48 *K. pneumoniae* 3/10 OXA-48 *E. coli*	Antibiotic consumption, high frailty, incontinence

CRKP, carbapenem resistant Klebsiella pneumoniae; PACF, post-acute-care facilities; CPE, carbapenemase-producing Enterobacterales; CRE, carbapenem-resistant Enterobacterales; LTCFs, long-term care facilities; MDR-GNB, multidrug-resistant micro-organisms; NA, not available; NHs, nursing homes.

As shown in **Table 3**, the prevalence rate of CRE colonization among LTCFs in the Asia region ranged from 13%–22.7% (Dandachi et al., 2016; Lee et al., 2017; Chen et al., 2018; Hagiya et al., 2018; Jean et al., 2018; Mao et al., 2018; Le et al., 2020). The two-point prevalence studies in Japan showed a 13% prevalence rate of CRE colonization in LTCFs in Hiroshima and a 19.3% prevalence rate in Osaka hospitals (one of which had approximately 200 long-term care beds) (Hagiya et al., 2018; Le et al., 2020). The CRE prevalence among the LTCFs in Taiwan was 22.7% (Lee et al., 2017). In Korea, a 10-month active surveillance survey *via* rectal specimens among LTCFs reported a low carbapenemase-producing Enterobacterales (CPE) prevalence rate (1.4%, 4/282) (Hwang et al., 2018). In Hong-Kong, no CPE fecal carriage was found in residential care homes for the elderly (RCHEs) (Dandachi et al., 2016), compatible with a previous study of 28 RCHEs in Hong-Kong by (Cheng et al. (2016).

**Table 3:** Studies of carbapenem-resistant Enterobacterales colonization in long-term care facilities in the Asia.

Authors	Study type	Sites	Study period	Study populations		Specimen sources	Sample size	Prevalence/incidence	LTCF percentage	Molecular studies	Risk factors
Le et al., 2020	Point prevalence study	Hiroshima (Japan)	2017/02 -2018/01		Residents in a LTCF	Oropharyngeal swab	98 residents in a LTCF	13% CRE	NA	1 MDR-*P. aeruginosa*. was a *bla*_IMP-1_ positive ST235	NA
Hagiya et al., 2018	Hospital admission	Northern Osaka (Japan)	2015/12 -2016/01		Admission to 43 hospitals	stool	140 patients from 43 hospital	19.3% CRE (27/140)	One hospital had 200 long-term care beds	IMP-6	Longer hospital stay, lower Norton scales
Lee et al., 2017	Point prevalence study	Taiwan	2015/01 -2015/12		Residents in 6 LTCFs	Rectal swab	313 residents in 6 LTCFs	22.7% CRE 11.7% CR-*K. pneumoniae* 5.2% CR-*E. coli*	NA	NA	Functional status, dementia
Chen et al., 2018	Point prevalence study	Hong-Kong	2015/09 -2015/12		20 RCHE	Nasal, axillary, rectal swab or stool	1,028 residents	0 in 373 stool and 654 rectal swab for CRE screening	NA	NA	NA
Mao et al., 2018)	Outbreak surveillance	Taiwan	2013/05 -2014/06		1 LTCF	Case 1 abscess Case 2 blood Case 3 blood, urine	4 isolates from 3 patients in a LTCF	NA	NA	4/4 *bla*_CMY-2_	NA

CRE, carbapenem-resistant Enterobacterales; IMP, imipenemase; LTCFs, long-term care facilities; NA, not available; RCHE, residential care homes for the elderly.

Collectively, the high predominance of CRE in LTCFs was observed worldwide, especially in LTCFs in the West region of the United States (California), Spain, Italy, Japan, and Taiwan. Since very few studies have been conducted to evaluate the clinical epidemiology of CRE in LTCFs in some regions in Europe (Russia, Arabia) and Asia (such as the mainland China, and the southern east countries), we could not determine the clinical epidemiology of CRE colonization and/or infections in these countries or regions.

### High-Acuity LTCFs Were an Important Reservoir of CRE

The high predominance of CRE in LTCFs reflects the local clinical epidemiology in the community or hospital settings, especially the ACHs or intensive care units (ICUs). In the following section, we compared recent studies evaluating CRE in LTCFs with previous studies about CRE in hospital settings and/or the community to demonstrate the issue.

In the United States, there were many studies supporting that the CRE acquisition and/or infections among residents of LTCFs was much more than that in acute care settings and/or the community. A population-based incidence study of CRKP among ACHs, LTACs, and SNHs in the Los Angeles County between 2010 and 2011 reported a higher CRE incidence rate in LTACs (2.54 per 1000 patient-days) than that in ACHs (0.31 per 1000 patient-days, p <0.001) (Marquez et al., 2013). Another outbreak investigation conducted among ACHs and LTACs in Indiana and Illinois in 2008 reported that one of the LTACs was the main locus of the outbreak, which accommodated 60% (24/60) of the cases, and only 10% (4/60) of the patients definitely had CRE colonization in ACHs (Gohil et al., 2017). A previous systematic review of CRE in the United States between 2000 and 2016 reported higher infection rates in LTACs than in ACHs and community settings (Livorsi et al., 2018), and community-onset cases mostly had health care exposure within the previous 90 days (Brennan et al., 2014). The multihospital case-control study in Chicago during an early KPC epidemic reported a higher prevalence of CRE carriage among LTCFs patients (8.3%, n=15) compared with patients admitted from the community (0%, n=0) (p<0.001) (Prabaker et al., 2012). Additionally, the HARP-DC studies (one of the first study to measure the prevalence of CRE colonization in a region aligning with CDC’s recommendation of collaborative approach), highlighted that the CRE prevalence in LTCFs was even higher than that in the ACHs (7.0% in LTCFs vs 5.0% in ACHs), with a relative prevalence ratio of 0.9% [0.5-1.5] in LTCFs and 1.5% [0.9-2.6] in ACHs) (Reuben et al., 2017). Furthermore, up to 30.4% prevalence rate of KPC-producing Enterobacterales among LTCFs was even reported (prevalence range 10–54%), compared with the relatively low prevalence rate in short-stay hospital ICU patients (3.3%, prevalence rate 0–29%) (Lin et al., 2013). That is, a 9-fold greater risk of KPC-producing Enterobacterales was found in the LTCF patients compared to the ACH patients (Lin et al., 2013). However, conflicting results of CRE prevalence between LTCFs and ACHs remain. A population based study conducted in Atlanta reported different results, of which CRE incidence was attributed mostly to the ACHs (n=141, 50%) and skilled nursing facilities (SNFs; n=113, 40%), rather than the LTACs (n=29, 10%) (Bower et al., 2020). A CRE incidence study by Guh et al. reported that most cases were collected from ACHs (33.9%) rather than from LTCFs (26.9%) or a LTAC (7.5%) (Guh et al., 2015).

In Europe, the European survey of carbapenemase-producing Enterobacterales (EuSCAPE study) evaluating CRE colonization and/or infections in hospitalized patients reported that CRE prevalence varied geographically, with the highest rate in the Mediterranean and Balkan countries (Grundmann et al., 2017). High incidence countries included Greece patients (5.78 per 10000 hospital admissions), Italy (5.96 per 10000 hospital admissions), Montenegro (5.65 per 10000 hospital admissions), Spain (4.01 per 10000 hospital admissions), and Serbia (3 per 10000 hospital admissions) (Grundmann et al., 2017). The high predominance of CRE in Spain and Italy were similar between ACHs and LTCFs, but the difference of CRE prevalence and/or incidence among LTCFs in other countries and/or regions in Europe had not been clarified in previous studies.

In Asia region, Kayoko Hayakawa et al. reported a 30% prevalence rate of carbapenemase-producing Enterobacterales (CPE) among tertiary hospitalized patients (Hayakawa et al., 2020), which was much higher than the CRE prevalence rate in LTCFs in another study in Japan (Le et al., 2020). However, Kayoko Hayakawa highlighted that patients with CPE were more likely to be residents in the nursing homes or the LTCFs prior to hospital admission (Hayakawa et al., 2020). Dokyun Kim et al. reported a low carbapenem resistance rate among *K. pneumoniae* in Korean secondary and tertiary hospitals (less than 0.1–2%), but an increasing trend of CRE (CRKP and *E. coli*) was reported in recent years (the carbapenem susceptibility rates of *E. coli* were 100% in 2011 and 99.3% in 2015; the carbapenem susceptibility rates of *K. pneumoniae* were 99.0% in 2011 and 97.0% in 2015) (Kim et al., 2017). In China, Qi Wang et al. conducted a longitudinal large scale CRE study between 2012 and 2016 among hospitals in China, and a high prevalence rate of carbapenem resistance among Enterobacterales was found (91% in *K. pneumoniae*, 80% in *E. coli*, and 72% in *E. cloacae*). However, there were few data about CRE prevalence in LTCFs in mainland China and other countries in Europe. Though little is known about the accurate difference of CRE prevalence between ACHs and LTCFs in the Asia region and Europe, studies in the United States, Italy, and Japan highlighted the threat of high prevalence of CRE among LTCFs, of which the prevalence and/or incidence of CRE acquisition and infections in LTCFs was much higher than that in ACHs, ICUs, and the community. Our review suggests that LTCFs are vital reservoirs for CRE and are important in regional outbreaks and/or dissemination of CRE (Chitnis et al., 2012; Prabaker et al., 2012; Lin et al., 2013; Guh et al., 2015; Gohil et al., 2017).

The CRE prevalence varied among different subtypes of LTCFs. One study reported a higher CRE prevalence in facilities that managed ventilated LTAC patients and ventilator-capable nursing homes (vNH) residents (8% *vs* < 1%, p<.001) and was rare in NHs that did not offer mechanical ventilation (NH <1%, vNH median 10% [0-12%], LTACs median 8%[8-10%]) (Mckinnell et al., 2019). Predominant CRE carriage among skilled nursing facilities with ventilator care (VSNFs) (27.3%) and LTACs (33.3%) (p <0.001) was found (Prabaker et al., 2012). Furthermore, patients from VSNFs and LTACs had a 7.0-fold greater odds ratio of KPC-producing Enterobacterales colonization (95% CI 1.3-42, p=0.022) than patients from SNFs (Prabaker et al., 2012). Collectively, high-acuity LTCFs that provided mechanical ventilation, such as ventilated LTAC, vNHs, and VSNFs, were particularly important for regional CRE spread.

### Similar CRE Species Distribution Between LTCFs and ACHs

The species distribution of KPC-producing Enterobacterales was similar between the ACHs and LTCFs in the United States, of which *K. pneumoniae* was the prominent species (Brennan et al., 2014; Guh et al., 2015; Cunha et al., 2016; Satlin et al., 2017; Jean et al., 2018; Hayakawa et al., 2020). The KPC-producing *K. pneumoniae* was the predominant species (87%, n=129), followed by *Enterobacter aerogenes* (6%), *E. coli* (4%), *E. cloacae* (0.7%), and co-colonization with *K. pneumoniae* plus either *E. coli* or *E. cloacae* (2.7%) (Lin et al., 2013). Consistent with the CRE incidence study from the United States communities in 2012–2013, a high prevalence (58.6%, n=351) of *K. pneumoniae* among CRE isolates was found among the United States population, followed by *E. coli* (13.2%, n=79) and *E. cloacae* (12.5%, n=75) (Guh et al., 2015). A high percentage of *K. pneumoniae* (87.2%, n=89) was also noted in a state-wide surveillance study in Michigan (Brennan et al., 2014).

In Europe, the predominant CRE species were also similar between the ACHs and the LTCFs. A hospital admission survey demonstrated *K. pneumoniae* as the primarily species in Spain, of which 37% of cases were health-care related (42% were NH residents) (Palacios-Baena et al., 2016). The EuSCPAPE study reported predominant *K. pneumoniae* among CRE species, followed by *E. coli*. Wide geographic variations in CRE prevalence existed in Europe, with a high prevalence in Italy, Romania, Turkey, and Spain (Grundmann et al., 2017).

In the Asian region, among CPE in Japan, the most common species were *E. cloacae* (30%), followed by *K. pneumoniae* (22%), *E. coli* (14.8%), *Citrobacter freundii* (11.1%), *Klebsiella oxytoca* (7.4%), *E. aerogenes* (3.7%), and *Serratia marcescens* (3.7%) (Hayakawa et al., 2020). The prominent CRE strains identified among LTCFs in Taiwan were *K. pneumoniae*, compatible with previous studies (Jean et al., 2018).

### Risk Factors for CRE Colonization and/or Infections Among Residents in LTCFs

Multiple risk factors were reported to be associated with the increased vulnerability for colonization and/or infections with CRE or specifically KPC-producing Enterobacterales. The identified risk factors could be broadly classified into four groups: patient characteristics, environmental factors (including facility subtypes and the use of medical devices) and previous microbiology status or antibiotic exposures (including previous hospital stay and co-infection with other pathogens) (**Table 4**).

**Table 4:** Risk factors for CRE acquisition in LTCFs.

Types of factors	Odds ratio or relative risks documented in studies
**Patient characteristics**	Fecal incontinence (OR 5.78) (Mills et al., 2016) Solid organ or stem cell transplantation (OR 5.05) (Mills et al., 2016) Immunosuppressive status (OR 3.92) (Bhargava et al., 2014) Comorbidities (Charlson’s score > 3; OR 4.85) (Bhargava et al., 2014) Strokes (Le et al., 2020), Dementia (Lee et al., 2017), Dependent functional status (Hagiya et al., 2018; Hayakawa et al., 2020)
**Environmental factors**	Usage of gastrointestinal devices (OR 19.7) (Cunha et al., 2016; Mckinnell et al., 2019) Indwelling devices (e.g. CVC or urinary catheters) (OR 5.21) (Lin et al., 2013) Mechanical ventilation (OR 3.56) (Mills et al., 2016) LTAC facility subtypes, esp. high-acuity facility with mechanical ventilation (Lin et al., 2013) Prolonged length of stay (Ben-David et al., 2011; Lin et al., 2013) Sharing a room with known carriers or increased prevalence of known carriers in the same ward (Chitnis et al., 2012)
**Microbiology status**	Prior antibiotic exposures (OR 3.89) (Chitnis et al., 2012; Bhargava et al., 2014; Brennan et al., 2014) Previous culture growing CRKP within 90 days (Chitnis et al., 2012) Recent *Clostridium difficile* infection (Prasad et al., 2016)

CVC, central venous catheter; CRKP, carbapenem-resistant Klebsiella pneumoniae; LTAC, long-term acute care hospitals; OR, odds ratio.

The identified patient characteristics associated with a significant risk factors for CRE colonization or infection among residents in LTCFs were fecal incontinence (OR 5.78, 95% CI 1.52 to 22.0, p=0.01) (Mills et al., 2016), solid organ or stem cell transplantation (OR 5.05, 95% CI 1.23 to 20.8, p=0.03) (Mills et al., 2016), comorbidity status with Charlson’s score greater than three (OR 4.85, 95% CI 1.64 to 14.41) (Bhargava et al., 2014), strokes (Le et al., 2020), dementia (Lee et al., 2017), residents in dependent functional status (Hagiya et al., 2018; Hayakawa et al., 2020), and immunosuppressive status (OR 3.92, 95% CI, 1.08 to 1.28) (Bhargava et al., 2014).

The environmental factors associated with a significant risk for CRE colonization or infection among residents of LTCFs were usage of gastrointestinal devices (OR 19.7, P <0.001) (Cunha et al., 2016; Mckinnell et al., 2019), mechanical ventilation (OR 3.56, 95% CI 1.24 to 5.28, p=0.01) (Mills et al., 2016), the presence of indwelling devices, such as central venous catheters or urinary catheters (OR 5.21, 95% CI 1.09 to 2.96), LTAC facility subtype (Lin et al., 2013), particularly high acuity facilities with mechanical ventilation, prolonged length of stay (Ben-David et al., 2011; Lin et al., 2013), and sharing a room with a known carrier (Chitnis et al., 2012).

Prior antimicrobial carriage status and associated antibiotics exposure were also associated with increased risk of CRE colonization or infection among residents in LTCFs (Ben-David et al., 2012). A high prevalence (41%) of residents in LTCFs had body cultures consistent with their prior CRE colonization status (Reuben et al., 2017). Prior antibiotic exposures (OR 3.89; 95% CI 0.71 to 21.47) (Chitnis et al., 2012; Bhargava et al., 2014; Brennan et al., 2014) and previous culture growing CRKP within 90 days were identified as independent risk factors for continued CRKP carriage (Chitnis et al., 2012). Furthermore, even short-term antimicrobial exposure in the prior one month was significantly associated with the increased risk of CRE colonization and infection among residents in LTCFs (meropenem OR 3.55, 95% CI 1.04–12.1, p=0.04; vancomycin OR 2.94, 95% CI 1.18–7.32, p=0.02; metronidazole OR 4.22, 95% CI 1.28–14.0, p=0.02) (Mills et al., 2016). In addition, recent *Clostridium difficile* infection was associated with increased risk of *C. difficile* and CRE colonization (Prasad et al., 2016), which indicated that prior or co-infection with other bacteria may increase CRE colonization risk in residents of LTCFs.

In the ACHs, the risk factors for CRE acquisition were exposure to antibiotics, high comorbidity indexes, deteriorated functional status and/or cognition at baseline, recent LTCF stay, and recent invasive procedures or permanent foreign devices (Lin et al., 2019). Our review of the risk factors associated with increased risk of CRE colonization and/or infection among residents in LTCFs was similar but not identical to the previous systematic review and meta-analysis (Van Loon et al., 2018), of which the systemic review identified risk factors associated with CRE acquisition among hospitalized patients between 2005 and 2017 in Europe, Asia, America, Australia, and Africa (Van Loon et al., 2018). Risk factors identified by Karlijn Van Loon et al. for CRE acquisition among hospitalized patients were patient’s underlying disease or condition (pooled OR 2.54; 95% CI 2.08 to 3.09, p <0.05), usage of medical devices (pooled OR 5.09; 95% CI, 3.38 to 7.67, p <0.05), mechanical ventilation (pooled OR 1.96; 95% CI 1.42 to 2.69, p<0.05), ICU admission (pooled OR 4.62; 95% CI, 2.46 to 8.69, p <0.05), antibiotic exposures (particularly carbapenem OR range 1.83 to 29.17 and cephalosporin OR 2.24 to 49.56), and CRE exposures (pooled OR 4.10; 96% CI, 1.46 to 11.52) (Van Loon et al., 2018).

### Resistance Mechanisms of CRE From LTCFs Were Similar to ACHs

**Figure 1:**
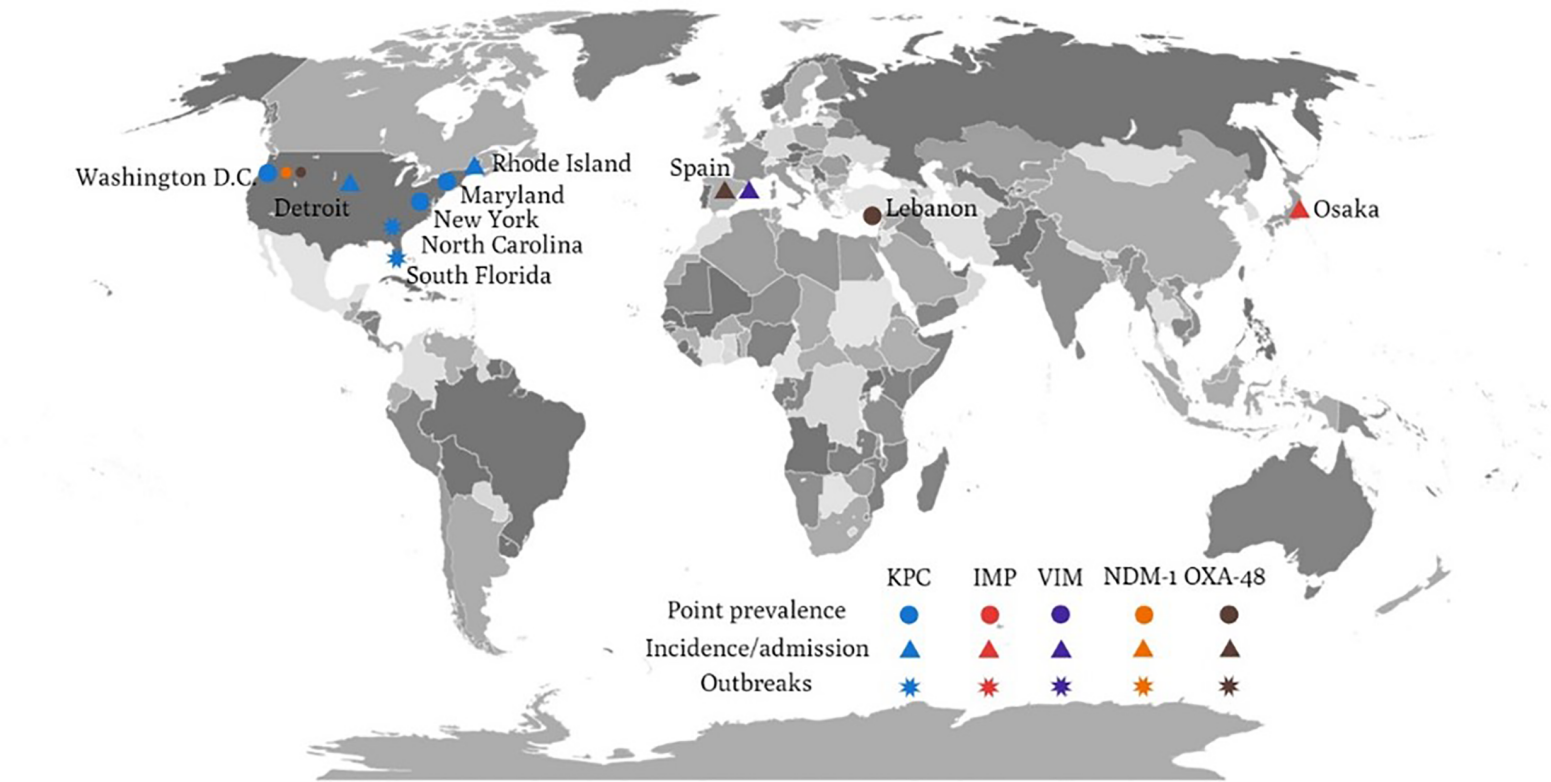
The global distribution of various carbapenemase-producing *Enterobacteriaceae* related to long-term care facilities.

KPC is the major resistance determinant of CRE from LTCFs in the United States (**Figure 1**). Molecular study for CRE from LTCFs in the United States demonstrated a high prevalence of KPC-producing Enterobacterales (55%) (Johnson et al., 2014). Among KPC-producing Enterobacterales, the prominent species were KPC-producing *K. pneumoniae* (87%), followed by *E. aerogenes* (6%), *E. coli* (4%), and *E. cloacae* (0.7%) (Lin et al., 2013). Among LTCFs in the United States, most KPC-producing *K. pneumoniae* strains carried KPC-2 (19/21) and mostly belonged to the strain ST258 (60%) (Johnson et al., 2014). Regarding KPC-producing *E. coli*, half of the tested strains belong to ST131 (Johnson et al., 2014). Another report showed a higher carriage rate of KPC-3 in *K. pneumoniae* and *E. coli*, whereas other carbapenemase (NDM, IMP, VIM, OXA-48) were uncommon among CRE colonized in LTCF residents in the United States (Prasad et al., 2016; Reuben et al., 2017; Dubendris et al., 2020). Among these studies, the carriage of CTX-M (Reuben et al., 2017) or OXA-48 (Reuben et al., 2017) could be found in KPC-producing Enterobacterales. The predominance of KPC-producing CRE isolates was supported by previous CRE incidence study, of which 90% KPC was identified from CRE (79.3% were *K. pneumoniae*) (Guh et al., 2015); and previous outbreak investigation study (Gohil et al., 2017). Furthermore, clustering of the ST258 was also noted in the outbreak investigation study in Indiana and Illinois (Gohil et al., 2017). Similarly, in the ACHs, the predominance of KPC-producing CRE (KPC-3 48%, KPC-2 44%) and co-existence of NDM-1 and OXA-48 were identified in a multicenter prevalence study in the United States (Satlin et al., 2017). In addition, the prominent ST258 strain among CRKP was also identified in ACHs (Satlin et al., 2017).

There are only few studies concerning the resistance mechanisms of CRE from LTCFs in Asia. Molecular analyses showed that nearly all CRE harbored IMP-type carbapenemase in LTCFs in Japan, of which IMP-11 was the most prominent type (40.7% IMP-11, 22.2% IMP-42, 14.8% IMP-6, 11.1% IMP-10, 11.1% IMP-1) (Hayakawa et al., 2020), though another study reported IMP-6 as the most common carbapenemase in Japan (Hagiya et al., 2018). Besides, efflux pump genes (*oqxA* and *oqxB*) were mostly observed in the CP-CRE group compared with the non-CP-CRE group (Hayakawa et al., 2020). A study in Taiwan revealed *bla*_CMY-2_ in imipenem-resistant *Providencia stuartii* isolates associated with the outbreak in a LTCF, yet the true determinant remained unidentified (Mao et al., 2018).

OXA-48 has been reported in CPE isolates from LTCFs in Europe. In a hospital admission survey of 379 CPE isolates from 245 patients in Spain, OXA-48 was the predominant carbapenemase, followed by VIM-1, IMP, and KPC (74% OXA-48, 22% VIM-1, 2% IMP, 2% KPC) (Palacios-Baena et al., 2016). In the 35 isolates from NHs, 32 of them were *K. pneumoniae*, of which 31 isolates were OXA-48 and one isolate was VIM-1. On the contrary, among hospitalized patients in Europe, KPCs remained the predominant carbapenemase (42%, 393/927), followed by OXA-48-like enzymes (38%, 353/927), NDM-1 (12%, 113/927), and VIM (7%, 68/927) (Grundmann et al., 2017). At country level, KPC were predominantly detected in Italy (96%, 187/195), Israel (80%, 31/39), Greece (65%, 56/86), and Portugal (59%, 36/61) (Grundmann et al., 2017). OXA-48-like carbapenemases were common in Turkey (79%, 98/124), Romania (74%, 50/68), Spain (70%, 81/116), Belgium (38%, 18/48), France (37%, 10/27), and Germany (33%, 12/36) (Grundmann et al., 2017).

### Outcome of CRE Colonization in LTCFs and ACHs

Acquisition of CRE is associated with a high economic burden and poor clinical outcomes. Among patients colonized or infected with CRE in LTCFs, the 30-day mortality rate was 10%–25% (Tischendorf et al., 2016; Chopra et al., 2018; Igbinosa et al., 2020). It is worth noting that the mortality rates in specific subgroups of patients with clinical CRE infections were as high as 30%–75% (Borer et al., 2012; Papadimitriou-Olivgeris et al., 2013; Lubbert et al., 2014). The condition is complicated by the prolonged CRE colonization which has been documented in various studies. Manon R. Haverkate et al. demonstrated that the duration of KPC-producing Enterobacterales colonization could be more than 9 months in KPC-positive patients (Haverkate et al., 2016).

The risk of infection after colonization with CRE varied in different studies. The cumulative risk of infection after CRE colonization was 16.5% in a systemic review (Tischendorf et al., 2016). The risk of infection varied, ranging from 7.6%–44.4% (Tischendorf et al., 2016), of which the most common site of infection was the lung (50% of the patients), followed by the urinary tract (20%), primary bloodstream, and skin and soft tissue infections (including surgical sites) (Tischendorf et al., 2016).

Regarding the hospitalized patients, a systemic review and meta-analysis revealed that the pooled risk ratio of CRE infection and mortality rate was 2.85 [95% CI, 1.88 to 4.30] (Soontaros and Leelakanok, 2019). Dickstein et al. conducted a matched cohort study in the ICU, and reported that colonization with CRE was independently associated with Enterobacterales infection (cause-specific hazard ratio was 2.06, 95% CI 1.31 to 8.43) (Dickstein et al., 2016). Zilberberg et al. conducted a retrospective cohort study among the hospitals in the United States, and reported that the presence of CRE was significantly associated with increased inappropriate empirical treatment than the absence of CRE (46.5% *vs*. 11.8%, p <0.001) (adjusted relative risk ratio 3.95, 95% CI 3.5 to 4.5, p <0.001) (Zilberberg et al., 2017). In addition, increased mortality rate (adjusted mortality 12%, 95% CI 3% to 23%) and prolonged length of hospital stay (an excess of 5.2 days, 95% CI 4.8 to 5.6, p <0.001) were found (Zilberberg et al., 2017).

Colonization with CRE poses an increasing threat to other residents in the same facility. CRE are mostly transmitted *via* patient-to-patient contact, and interestingly, the CRE transmission in the environment follows the 20/80 rule. That is, 20% super-spreaders are responsible for 80% bacterial transmission, which indicated that the super-spreaders play an important role in CRE transmission (Lerner et al., 2015). CRE super-spreaders were associated with high rectal CRE concentrations (modelled as *bla*_KPC_ copies/16s rDNA copies ratio, OR 14.5, 95% CI, 1.09 to 192.0, p=0.042) and respiratory illnesses on admission (OR 20.5, 95% CI, 1.41 to 297.6, p=0.027) (Lerner et al., 2015).

### The Consideration of Active Surveillance for CRE in LTCFs

In 2016, with the worsening threat of CRE, the European Centre for Disease Prevention and Control (ECDC) recommended infection prevention and control measures in hospitals and other healthcare settings, active screening in the epidemic region, and active surveillance in the endemic region. Even though active surveillance is one of the infection control measures for the prevention of CPE transmission and spread (Ambretti et al., 2019), and the 2013 European Society of Clinical Microbiology and Infectious Disease (ESCMID) strongly recommended contact precautions, using alert codes to identify known colonized patients at admission, pre-emptive contact precautions, isolation in a single room for infected or colonized patients, cohort staffing, antimicrobial stewardship and education, monitoring cleaning performance and active surveillance (Ambretti et al., 2019); there are limited national or international guidelines for optimal measures for active surveillance and management of CRE colonized patients in LTCFs (Ambretti et al., 2019). Some experts suggested that screening all hospital admission from the LTCFs for CRE may not be cost-effective (Hwang et al., 2018).

For CRE, targeting patients at high risk of CRE carriage is very important, and these high risk patients should be screened for digestive tract carriage, with concomitant pre-emptive contact precaution and followed isolation if colonization was confirmed (Magiorakos et al., 2017). Lau A. F. et al. recommended that the universal nucleic acid amplification technology (NAAT)-based method of CRE screening may not be universally affordable, but molecular rapid methods may be applicable for high-risk patients (e.g. from endemic region, LTCFs, extensive exposure to carbapenems), followed by susceptible culture-based method if the screening is negative, and considering to perform the carbapenemase confirmatory test if suspicious species are detected (Lau et al., 2015). Other than molecular and/or genomic analyses, Vincent J. LaBombardi et al. suggested that Remel Spectra CRE agar plates could provide faster (18 hours for Remel Spectra CRE agar versus 36 hours for the CDC method) and reliable results for KPC-type CRE detection (Labombardi et al., 2015). The sensitivity for common commercial chromogenic media were also reported, including chromID Carba media 96.5%, Remel Spectra CRE 97.8%, CHROMagar KPC 76.6%, and Direct ertapenem disk method 83% (Labombardi et al., 2015).

The CDC recommended perirectal swab for targeted CRE screening at hospital admission, especially for those admitted directly from LTACs, other LTCFs with known endemicity, or patients who are transferred directly from another ACHs (Tischendorf et al., 2016). Although KPC-producing Enterobacterales were mostly identified in perianal swab specimens (Johnson et al., 2014) or rectal swab culture (Cunha et al., 2016)), axilla/groin screening samples also detected 1% CRE prevalence in nursing home facilities (Mckinnell et al., 2016). CRE could be found in a variety of samples. Various studies reported urine as the most common source of CRE (Brennan et al., 2014; Guh et al., 2015), and CP-CRE were prominently isolated from sputum (40.7%) (Hayakawa et al., 2020). In a study conducted in Hiroshima, CRE were detected in oropharyngeal swab specimen (Le et al., 2020), and in a study in Osaka, CRE were detected from blood, sputum, urine, and intra-abdominal samples (Hayakawa et al., 2020).

### Interventions to Reduce CRE Colonization and Infection in LTCFs

The importance of interventions to reduce the CRE carriage rate in LTCFs has already been documented. An outbreak investigation of CRE colonization among patients in LTAC between 2009 and 2011 revealed that surveillance testing and targeted interventions resulted in significant reductions in CRE prevalence (49% *vs* 8%), CRE incidence (44% *vs* 0%) and CRE bacteremia (2.5 *vs* 0.0 per 1000 patient-days) (Chitnis et al., 2012). In addition, the prevention measures for CRE in LTCFs from a nation-wide coordinated protocol in Israel between 2007 and 2008 successfully reduced nosocomial CRE cases from 55.5 cases per 100000 patient-years to 11.7 cases per 100000 patient-years (Schwaber et al., 2011). The infection prevention measures in Israel were contact isolation, self-contained nursing units (single room or cohorts), and re-isolation of known carriers when they are encountered for subsequent hospital admission (Schwaber et al., 2011). Furthermore, the subsequent nation-wide intervention (alcohol-based hand rub, appropriate use of gloves, and a policy of CRE surveillance at hospital admission) in 13 post-acute hospitals in Israel between 2008 and 2011 resulted in the reduction of the overall CRE carriage rate (16.8% vs 12.5%, p=0.013) (Ben-David et al., 2014). Collectively, the Israeli guidelines for CRE prevention were isolation or cohorting of CRE carriers, cohorting of nursing staff (mandatory only for ACHs), barrier precautions on room entrance, admission CRE screening for high-risk patients, screening of patients’ contacts, standard protocol for discontinuation of contact isolation, daily reporting of new cases to the Israeli National Center for Infection Control in the Ministry of Health, and periodic auditing of health care facilities’ compliance with national guidelines by the National Center for Infection Control in the Ministry of Health (Ben-David et al., 2014).

In the United States, Mary K. Hayden et al. (Hayden et al., 2015) examined a bundled intervention in four LTACs in a high endemic KPC-producing Enterobacterales region (Chicago, Illinois) (Hayden et al., 2015). They adopted a rectal swab culture for active surveillance and preemptive contact isolation while awaiting the culture report. KPC intervention bundle was as follows: biweekly rectal culture surveillance, contact isolation, geographic separation of KPC-positive patients with ward cohort or single room, universal contact isolation of all patients in a high-acuity ward, daily 2% chlorhexidine gluconate (CHG) bathing for patients, healthcare-worker education, and adherence monitoring (particularly hand hygiene) (Hayden et al., 2015). During the pre-intervention period, the prevalence rate of KPC-producing Enterobacterales remained unchanged (average 45.8%, 95% CI 42.1% to 49.5%) (Hayden et al., 2015). During the intervention period, the prevalence rate of KPC-producing Enterobacterales declined significantly, then reached a plateau (34.3%, 95% CI 32.4 to 36.2%, p <0.001) (Hayden et al., 2015). The incidence of KPC-producing Enterobacterales also declined significantly during the intervention (from 4 to 2 acquisition per 100 patient-weeks, p=0.004) (Hayden et al., 2015). Other important clinical outcome indicators, including KPC identified in any clinical culture (32% reduction) and KPC bacteremia (56% reduction) also decreased (Hayden et al., 2015).

Similar to Hayden et al.’ s study (Hayden et al., 2015), Toth et al. designed an agent-based simulation model for CRE transmission in a single LTAC within a regional network of ten health-care facilities (including one LTAC, three ACHs, and six NHs). The CRE prevalence rate was 45.8% and clinical detection incidence was 3.7 per 1000 patient-days (Toth et al., 2017). Two models with different transmission rates were created, and significant reduction in CRE transmission (range 79% to 93%) and prevalence (decrease from 21% to 6% in model A, decrease from 9% to 0.5% in model B) in a five-year period were reported (Toth et al., 2017). Even when the intervention was delayed until the 20^th^ CRE detection, CRE transmission was still reduced by 60%–79% over five years (Toth et al., 2017). Furthermore, among the infection control measures, Toth et al. (Toth et al., 2019) designed a model-based assessment in Chicago, and reported that contact precautions by itself could potentially explain the decline in CPE colonization among surveillance-detected carriers (Toth et al., 2019). Other interventions, including CHG bathing, hand hygiene, and adherence monitoring may play a role in the slowing down of colonization (Toth et al., 2019). Therefore, focusing infection control and prevention measures in LTCFs is an effective strategy to reduce CRE acquisition and transmission (Toth et al., 2017).

Moreover, the importance of controlling CRE in LTCFs could not be over emphasized, since it may become a tragedy without interventions. Bruce Y Lee et al. conducted a prediction model and reported that if infection control and prevention measures were not implemented properly, CRE would become endemic in almost all health-care facilities (in Orange County) within 10 years (Lee et al., 2016). Although benefits of CRE decolonization include reduced CRE-related infection incidence and all-cause mortality (Tacconelli et al., 2019), the potential of increased antimicrobial resistance to decolonizing agents was reported in nearly all studies (Tacconelli et al., 2019). Consequently, the ESCMID-EUCIC clinical guidelines do not recommend routine decolonization of CRE (Tacconelli et al., 2019).

Antimicrobial use in LTCFs remains a critical issue in long-term care (Ricchizzi et al., 2018). Antimicrobial stewardship programs are needed to control the spreading of multidrug-resistant organisms (Oliva et al., 2018). Current data suggested effective antimicrobial stewardship strategies in LTCF reduced antimicrobial use (Jump et al., 2012). The implementation strategies vary considerably in different setting and warrant more studies to define appropriate and quality appraisal tools (Wu et al., 2019).

### Summary

The emergence of CRE has become a major public health concern. Moreover, its colonization among residents of LTCFs is associated with subsequent infections and mortality. LTCFs are also an important reservoir of CRE. There are increasing studies concerning the high predominance of CRE in LTCFs. Further studies are needed to develop effective control measures.

## Author Contributions

H-YC, P-YL, S-SJ, Y-LL, M-CL, W-CK and P-RH collected and analyzed the data. S-SJ, Y-LL, M-CL, and W-CK conducted validation and supervision. H-YC, S-SJ, Y-LL, M-CL, W-CK and P-YL participated in the writing of the manuscript. H-YC, P-YL, S-SJ, Y-LL, M-CL, W-CK and P-RH read and approved the final version of the manuscript. All authors contributed to the article and approved the submitted version.

## Conflict of Interest

The authors declare that the research was conducted in the absence of any commercial or financial relationships that could be construed as a potential conflict of interest.
